# Preliminary Study on Microplastic Contamination in Black Sea Cetaceans: Gastrointestinal Analysis of *Phocoena phocoena relicta* and *Tursiops truncatus ponticus*

**DOI:** 10.3390/ani14060886

**Published:** 2024-03-13

**Authors:** Adrian Filimon, Andreea-Mădălina Ciucă, George-Emanuel Harcotă, Elena Stoica

**Affiliations:** 1 National Institute for Marine Research and Development “Grigore Antipa”, 300 Mamaia Blvd., 900581 Constanta, Romania; aciuca@alpha.rmri.ro (A.-M.C.); gharcota@alpha.rmri.ro (G.-E.H.); 2 Doctoral School Biotechnical Systems Engineering, University POLITEHNICA of Bucharest, 060042 Bucharest, Romania

**Keywords:** cetaceans, gastro-intestinal tract, plastic, microplastics, marine litter, ingestion, contamination, Black Sea, Romania

## Abstract

**Simple Summary:**

This study addresses the microplastic contamination in the gastro-intestinal tract (GIT) of two cetacean species along the Romanian coast. Microplastic contamination in cetacean GITs was very little researched in the Black Sea. The GIT content of stranded and by-caught cetaceans was processed using a multi-sieve tool, an innovative approach never applied to the Black Sea. After sieving, the samples underwent a laboratory treatment to minimize organic materials in the samples, facilitating the observation of plastics under a stereomicroscope. All investigated individuals had ingested plastics, especially microplastics. Compared to other studies, the number of microplastics found was much higher. One hypothesis that these data point to is that the Black Sea may be more polluted with plastic litter than other European seas. To test this hypothesis, a more extensive analysis involving a larger number of samples should be undertaken. Simultaneously, intensifying research efforts could contribute to a more comprehensive assessment of the marine environmental status, under Marine Strategy Framework Directive (MSFD).

**Abstract:**

Plastic pollution is a global concern that has a significant impact on marine life. Plastic is widely used and has become a pervasive pollutant in marine environments. Plastic contamination has been documented both in marine environments and biota. Plastic contamination in cetacean gastro-intestinal tract (GIT) content has received limited attention, especially in the Black Sea. This study aims to investigate plastic contamination in the GITs of bottlenose dolphins and harbour porpoises, introducing a novel methodology. Given the limited exploration of this issue in the Black Sea, the research predominantly focuses on microplastic contamination. The GITs were sampled through necropsy from stranded and by-caught cetaceans, and content was washed through a multi-sieves tool. The material retained on each sieve was analysed following specific protocols. All (100%) of the GITs contained plastics (meso- and microplastics). In total, 1059 items (fibres, fragments, and beads) ranging from 22.86 µm to 5776 µm were found, suggesting a high contamination level in the Black Sea cetaceans. Future efforts should concentrate on increasing the number of samples and using the results for the implementation of the Marine Strategy Framework Directive (MSFD).

## 1. Introduction

Due to its versatile qualities, plastic is extensively used in a wide range of products [1,2]. However, it has become a pervasive environmental pollutant, categorized into macroplastics (>2.5 cm), mesoplastics (0.5–≤2.5 cm), large microplastics (1000–≤5000 µm), small microplastics (1 µm–≤1000 µm), and nanoplastics (1 nm–≤1 µm) [3]. The slow decomposition of plastic ensures its long-lasting presence in the environment [1]. Both marine and land-based sources contribute significantly to approximately 80% of plastic debris present in marine environments [1]. The severity of marine plastic litter has forced global acknowledgement, resulting in the formulation of official strategies in response to its harmful impact on wildlife and seafood [4].

In addition, the European Union recognizes marine litter as a critical problem and has integrated it into environmental policies, such as the Marine Strategy Framework Directive (MSFD) 2008/56/EC. This directive is essential for assessing the state of the environment of marine ecosystems, via its qualitative descriptors, notably Descriptor 10 (D10), concerning marine and plastic litter, focusing on the reduction and prevention of litter in European marine waters. Under D10, the ingestion of litter by marine animals (i.e., birds, mammals, reptiles, fish, or invertebrates) is also assessed. The objective of this assessment is to ascertain whether the quantity of litter and micro-litter consumed by marine species remains at levels that do not adversely affect their health [5].

Marine litter is a pollution problem affecting thousands of marine species primarily due to ingestion and entanglement [6,7,8,9,10,11]. Cetaceans are also adversely affected by plastic pollution. Plastics and other marine debris have been found in the gastro-intestinal tracts of cetaceans, likely causing impairment to digestive processes and even death [12].

The Black Sea, which is surrounded by six countries, is at an increased risk due to the significant discharge of rivers into this semi-enclosed basin. This amplifies the threat of marine litter and plastic pollution, as indicated by various studies [13,14,15,16,17,18,19].

The Black Sea has low marine mammal biodiversity, with only three subspecies of cetaceans: the common dolphin (*Delphinus delphis ponticus* Barabash, 1935), the bottlenose dolphin (*Tursiops truncatus ponticus* Barabash-Nikiforov, 1940), and the harbour porpoise (*Phocoena phocoena relicta* Abel, 1905). All three species are included in the IUCN Red List and are listed in annexes II and IV of the EU Habitats Directive 92/43/EEC in EU waters. Additionally, they are classified under Descriptor 1 (D1—Biodiversity) of MSFD.

The pollution caused by marine debris poses a severe threat to the cetacean species in the Black Sea. The presence of macroplastics and other human-made debris in the GIT of Black Sea cetaceans has been documented before [20,21,22,23,24].

The interaction between cetaceans and microplastics is a matter of great concern. Cetaceans interact with microplastics through direct ingestion from the environment or through trophic transfer. Although there are several studies available for other regions [25,26,27,28,29,30], in the Black Sea, the topic is still largely unexplored. The lack of information is mostly attributed to limited research endeavours. The present study aimed to fill this knowledge gap by examining plastic (especially microplastic) contamination in the GIT of Black Sea cetaceans using an innovative approach [31].

Our research methodology and the data collected are fully in compliance with MSFD, and the findings of the investigation could be a foundational step for employing it in marine litter monitoring efforts in the Black Sea region.

## 2. Materials and Methods

### 2.1. Study Area

**Figure 1:**
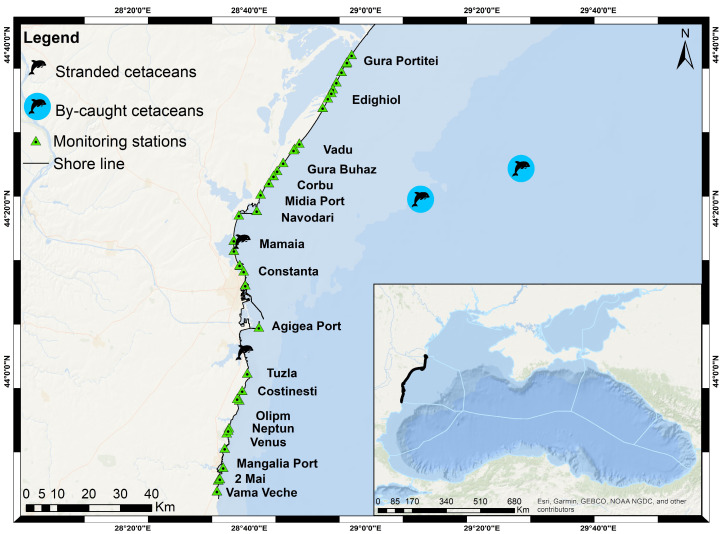
Map indicating study area.

The study was conducted in the northwestern Black Sea, specifically on the Romanian shelf (Figure 1). The Romanian Black Sea coastline extends for over 240 km, divided into northern and southern sectors. The northern sector, approximately 158 km in length, stretches between the secondary delta of the Chilia branch and Constanta and is constituted of alluvial sediments. The shallow waters, up to a depth of 40 m in this sector, are included in the Biosphere Reserve of the Danube Delta. The southern sector, around 85 km in length, extends between Constanta and Vama-Veche, is characterized by promontories with active, high cliffs, and is separated by large zones with accumulative beaches that often protect littoral lakes. The distance from the seashore to the shelf limits (200 m depth) varies from 100 to 200 km in the northern sector to 50 km in the southern one. The Romanian coast is mainly influenced by the northern sea waters because of the north-south general current system [32]. 

### 2.2. Post-Mortem Investigation

In 2023, the National Institute for Marine Research and Development “Grigore Antipa” (NIMRD) team conducted monitoring campaigns for stranded and by-caught cetaceans. Harbour porpoises and bottlenose dolphins found stranded and by-caught in turbot gillnets were gathered for necropsies. Essential data for each cetacean, including date and location, overall length, weight, sex, age, and decomposition condition code (DCC), were recorded. The carcasses of stranded cetaceans were examined for any external lesions and any external signs of fishery interaction. The necropsies and GIT sampling were carried out at the NIMRD laboratory. All necropsies were executed following the ACCOBAMS and ASCOBANS best practice protocol [33]. The GIT was collected from cetaceans within the DCC 1–4 only if it was intact otherwise the results could be compromised.

In total, 4 cetaceans met the conditions for GIT sampling (Table 1).

Before collection, the GITs were sealed at both ends to minimize the contamination from environmental sources and frozen at −20 °C until processing. 

### 2.3. GIT Content Processing 

The sampling of GIT content was made according to Corazzola et al. [31]. Briefly, the GIT sections (i.e., stomach, and intestine) were individually washed using a multi-sieve tool. The process of sieving GIT content has proven to be highly effective compared to other methods [31,34]. Before sieving, the GIT was carefully rinsed with tap water to remove any blood and other particles that could potentially affect the quality of the samples. The stomach was separated from the intestines, and seals (i.e., white serrated band made of nylon PA66) were removed from both cranial and caudal ends. The stomach chambers and intestines were individually dissected using metal scissors on the uppermost sieve of the multi-sieve tool and then gently rinsed with tap water. The content was washed with tap water through 5000 µm, 1000 µm, 500 µm, 250 µm, and 100 µm mesh sizes.

### 2.4. Sample Analysis

During sample analysis, a range of foreign objects, including stones, sand shell fragments, plants, plastics, and other human-made debris, were noticed and recorded. This study especially focused on microplastics and other anthropogenic debris. The protocol provided by Lusher et al. [35] was followed to process samples for plastics analysis. To decompose the organic (non-plastic) components in the samples, a solution of KOH (10%) was added to the samples in a ratio of 3:1, and the mixture was subjected to incubation at a temperature of 60 °C for 24 h. Following digestion, the samples underwent vacuum filtration using 1.6 µm glass fibre filters in a fume hood. Subsequently, the filters were left to dry in covered glass petri dishes. For samples containing sand, a prefiltration step was employed to separate plastics. The sorting method involved a saline solution with a density of 1.2 g/cm^3^ and a separatory funnel. The separatory funnels were well shaken and left to separate for 2–12 h, depending on the amount of material to be separated. The filters were visually inspected under an Olympus SZX10 microscope foreseen with an SC50 camera. The identification of plastic items was conducted according to the criteria proposed by Lusher et al. [36]. The measurement of plastics was made using cellSens Entry software Version 1.16. Then, a needle heated until it reached a high temperature was carefully applied to all particles. If the particle melted or deformed, then it would suggest the presence of plastic.

### 2.5. Contamination Control and Procedural Blanks

Strict protocols were enforced during the sample collection and laboratory processing phases to avoid contamination. Before extraction, the GIT was sealed using a white serrated band made of nylon PA66 at the cranial and caudal portions of the stomach and intestine to minimize the contamination of GIT content from environmental sources of microlitter items and to avoid the mixing of the content. In the laboratory, all tools and glassware were thoroughly rinsed with distilled water and ethanol (70%) and stored in aluminium foil. Ethanol (70%) was used to clean the surfaces and equipment in the laboratory. Nitrile gloves and white cotton lab coats were worn during necropsies and laboratory analyses. During GIT sampling and sample analysis, access to the laboratory was restricted. When not in use, samples were always wrapped in aluminium foil. The materials and tools used were made of glass and stainless steel. 

To assess contamination, procedural blanks (*n* = 11) and controls (*n* = 4) were taken multiple times. For procedural blanks, before GIT content sampling, 500 mL of water was run into the support and into the 500 μm, 250 μm, and 100 μm sieves to capture any microlitter items that may be present in the device. The water was sampled in pre-cleaned glass jars and analysed for microplastics [31]. Controls, represented by ultrapure water blanks and glass microfibre filters, were kept in the working environment during the whole processing (i.e., opening and rinsing the intestines, sample processing, and observation and identification under the stereomicroscope) to collect the microlitter items present in the air. All procedural blanks and controls followed the same treatment as all samples. The microplastic particles found were examined under a stereomicroscope, where they were counted, and details regarding their type, colour, and size were recorded. Subsequently, an equivalent number of particles with matching characteristics were systematically removed from the overall database, maintaining a 1:1 ratio subtraction ratio.

### 2.6. Data Analysis

The raw data were initially introduced in Excel, where preprocessing and organisation took place. The results included numerical data on the plastic litter. The first analysis in this study focused on frequency of occurrence (%FO). The one-way analysis of variance (ANOVA) was applied using statistical computing software R Version 4.3.2. In the ANOVA analysis, the statistical differences in the data set corresponding to different GIT sections (i.e., stomach and intestine) were evaluated. 

## 3. Results

This study examined a total of eight digestive organs, consisting of four stomachs and four intestines, collected from three *T. t. ponticus* and one *P. p. relicta*. All animals were adults, three males and one female. The analysis revealed the presence of synthetic particles in all of them, resulting in a frequency of occurrence (FO%) of 100%.

**Figure 2:**
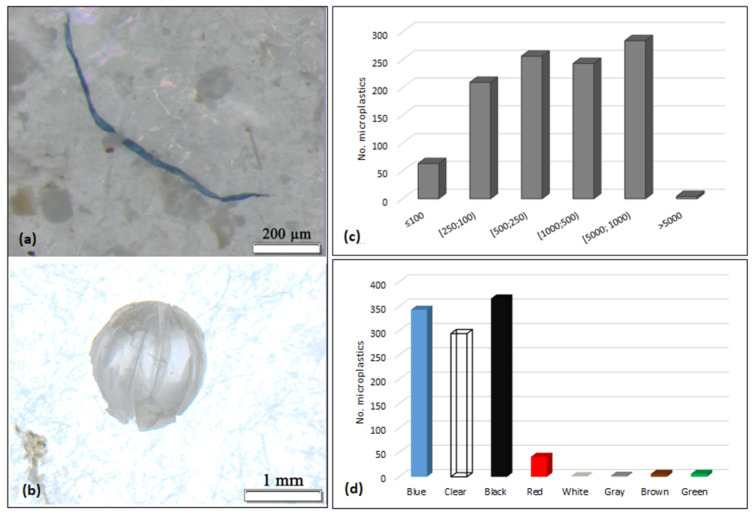
Microplastic general information: (**a**) blue fibre (scale 200 µm); (**b**) clear bead (scale 1 mm); (**c**) dominant sizes; (**d**) dominant colours.

A comprehensive investigation indicated a cumulative total of 1055 microplastics and four mesoplastics, with individual counts ranging from 119 to 388 particles per organism. Out of the total, 91.78% (*n* = 972) were classified as fibres, 8.12% (*n* = 86) were categorized as fragments, and those remaining (0.09%; *n* = 1) were represented by a spherical bead (Figure 2a,b).

The most prevalent microplastics (27%; *n* = 284) were those with sizes ranging from 5000 to 1001 µm. They were followed by microplastics measuring between 500 and 251 µm (24%; *n* = 256), 1000 and 501 µm (23%; *n* = 243), 250 and 101 µm (20%; *n* = 209), and ≤100 µm (6%; *n* = 63) (Figure 2c). In each GIT, a single mesoplastic item (i.e., >5000 µm) was found.

The fibres varied in size, ranging from 22.86 µm to 5776 µm, with an average length of 957.20 µm (±920.65 SD). The fragments exhibited a size range from 25.57 × 13.19 µm to 2184.38 × 515.89 µm, with an average dimension of 417.06 µm (±478.42 SD) × 172.97 µm (±138.67 SD).

Regarding particle colour, a total of eight distinct colours were identified in the samples (Figure 2d). The predominant colours were black (34%; *n* = 366), blue (32%; *n* = 343), and clear (28%; *n* = 294). The other encountered colours (i.e., red, white, grey, brown, green) comprised a cumulative proportion of 6% (*n* = 56).

**Figure 3:**
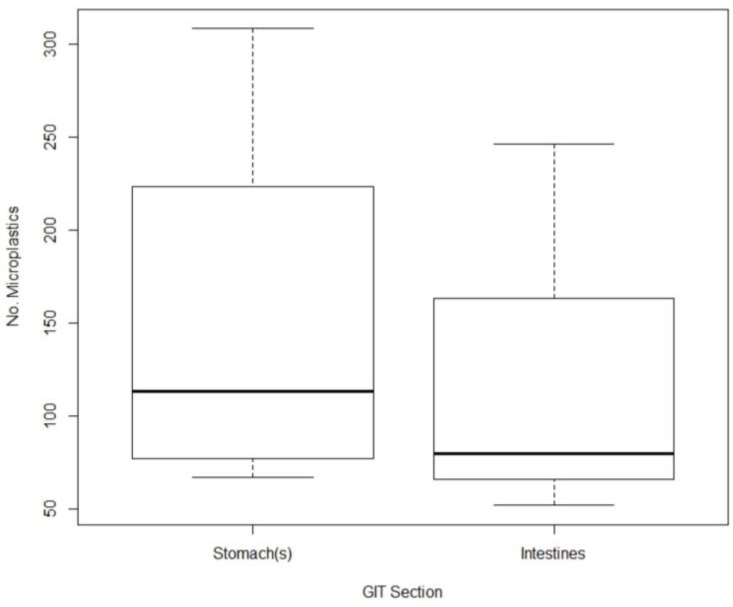
Boxplot showing the median number of microplastics in the intestine and stomach.

Among the GIT sections, the number of microplastics was higher in stomachs (*n* = 599; mean 149.75 ± 109.9677 SD) compared to intestines (*n* = 456; mean 114 ± 89.1291 SD) (Figure 3). The stomachs contained a greater number of both small (1 µm–≤1000 µm) and large microplastics (1000–≤5000 µm) [3], with 419 small and 180 large particles, compared to the intestine, which contained 352 small and 104 large microplastics. Nevertheless, the one-way ANOVA analysis did not show a statistically significant difference in the number of microplastics among the GIT compartments (Pr(>F) = 0.984, *p* = 0.6298).

In terms of environmental contamination, the study adhered to EU guidelines, which specify that background contamination with microplastics should not exceed 10% of the overall average of microplastics found within all analysed samples [37]. In the procedural black and controls, the contamination was 6% (*n* = 18) of the overall average microplastics found. Of the 18 particles found, 14 items were found in the procedural blank, and 4 items in the controls. Microplastic items with the same characteristics as the items found on the blanks were excluded from the database [30].

## 4. Discussion

This is the first study to investigate the microplastic contamination in the GIT content of cetaceans in Romania and the second scientific inquiry within the Black Sea region. Additionally, it introduces a novel methodology to the Black Sea, employing a multi-sieve tool for the simultaneous assessment of ingested macro-, meso-, and micro-litter across all distinct sections of GIT [31]. 

Broadly, our observations align with the outcomes of prior research. In our investigation, we found microplastic particles in all the analysed samples. A frequency of occurrence (FO%) of 100% is in line with most of the available studies [30,35,38,39,40,41]. 

The quantity of plastics documented in this study (1059 plastics, including 1055 microplastics) was notably higher than reported in studies conducted in other marine regions. For instance, analyses of the entire gastro-intestinal tracts (i.e., stomachs and intestines) of five cetaceans stranded on the Italian coast using the same methodology for sample processing revealed the presence of only 173 plastic items, including 161 microplastics [31]. Another study involving 38 stranded cetaceans on the Portuguese coast documented 268 plastic items (254 microplastics) [30]. Similarly, in the digestive tract of 43 striped dolphins stranded on the Mediterranean coast of Spain, a total of 672 plastic items were reported [41]. On the British coast, investigations of the stomachs and intestines of 50 marine mammals (43 cetaceans) identified 273 plastic particles (including 261 microplastics) [38]. The abovementioned studies reported the prevalence of microplastics ingested by cetaceans, which is similar to our findings. Meso- and macroplastics were either present in low numbers or absent. Comparative data on plastic contamination in GIT content for the Black Sea are very limited in availability. The only currently available study revealed that 84% of 31 cetaceans had ingested plastics, and a total of 197 plastic particles were found [42]. Because of the large variation in the number of microplastic items identified in the two studies, the comparison needs to be carefully considered. Anyway, in terms of ingested plastic quantity, comparisons between studies are challenging because of differences in GIT compartments analysed and the methodology followed [30]. In addition to the already mentioned factors, there could be other variables that can influence the number of ingested microplastics [38]. 

Our research showed that fibres were the most common type of microplastic ingested. The fibre prevalence was claimed in most of the published studies [26,35,39,41,43]. While investigating the translocation of microplastics in organs, the dominance of fibres was also reported in the lung tissue, melon, acoustic fat pad, and blubber [44]. Studies conducted in the Black Sea region on biota and environmental matrices have also reported the predominance of fibres [17,45,46,47]. The Black Sea’s microplastics may originate from river and urban runoff, industrial discharges, and the disintegration of larger debris [48,49,50,51]. Fibres could be a result of industrial discharges, whereas fragments are the result of the degradation of bigger plastic products. A study showed that ropes and nets (made of polypropylene, polyethylene, and nylon) used in fishing operations are an important source of fibres [52].

Studies generally indicate a variety of colours of microplastics, ranging from blue to transparent. A comprehensive review of articles focusing on microplastic ingestion in marine biota unveiled that blue (32.94%), white (24.71%), black (18.82%), and transparent (16.47%) are the most prevalent microplastic colours encountered. The most common colours found in marine mammals were blue (50%), transparent (37.5%), and black (12.5%) [53]. The black and blue colours were demonstrated to be prevalent both in the Black Sea environment and in biota [54]. Additionally, there is evidence that some species of fish often ingest blue microplastics by mistake, as they resemble their natural prey such as the blue pigmented copepods *Pontella sinica*, *Sapphirina* sp., or *Corycaeus* sp. [55]. Black and blue were the most common colours in our study, which are comparable to the colours that are the most frequent in cetaceans, as documented by Zantis et al. [56]. Certainly, an important source of blue fibres could also be attributed to fishing activities, as the colour blue is commonly used for ropes and nets.

The results of the present study showed that the stomachs of the cetaceans contained more microplastics than the intestines. Some scientists suggest that these differences can be because the stomachs of cetaceans may act as a reservoir for the accumulation of plastic in GIT [38]. On the other hand, some studies have shown the existence of microplastics throughout the entire intestine increasing the likelihood that they may be excreted [25,28]. The finding of microplastics in the scats of various marine mammal species, including *Halichoerus grypus*, *Arctocephalus* spp., and *Callorhinus ursinus*, supports this hypothesis [57,58,59]. Due to divided opinions among scientists, further research is needed to validate these assertions. 

Microplastics have been discovered in the digestive tracts of zooplankton, which is an intermediary in the food chain [19]. This suggests that, due to their smaller size, microplastics may be able to move up the food chain from lower trophic levels to higher ones, where they may eventually end up in fish, birds, turtles, and marine mammals. Microplastic contamination has been documented in pelagic and benthic fish species (e.g., *Engraulis encrasicolus*, *Trachurus mediterraneus*, *Sarda sarda*, *Belone belone*, *Pomatus saltatrix*, *Merlangius merlangus*, and *Mullus barbatus*), the prey of the cetaceans living in the Black Sea, revealing a high contamination rate [19,47]. 

There are different ways for cetaceans to ingest microplastics (i.e., direct ingestion from the environment or through trophic transfer). The degree to which microplastics are internalized through direct ingestion from the environment is currently unknown [35]. However, we agree with the other statements emphasizing the crucial role of feeding in plastic ingestion [38]. Black Sea cetaceans are raptorial feeders that use teeth to catch prey and are more likely to ingest plastic items through trophic transfer [60]. As preferred prey, harbour porpoises exhibit a preference for gobies, whereas bottlenose dolphins show a preference for turbots and mullets. Additionally, it is recognized that Black Sea cetaceans undertake mass migrations to the north in spring and to the south in autumn generally associated with the movements of pelagic fish stocks, particularly anchovies. Both harbour porpoise and bottlenose dolphin eagerly eat anchovies, especially when they occur in large and dense schools. The findings of a recent study on microplastic contamination in Black Sea fish species revealed 233 plastic particles (including 157 fibres) in the GIT of 335 anchovies (*Engraulis encrasicolus*), as well as 59 plastic particles (including 38 fibres) in 155 red mullets (*Mullus barbatus*) [47]. Considering that the estimated weight of 335 anchovies is approximately 2.5 kg, and a harbour porpoise can consume between 3 and 5 kg of fish per day, while a bottlenose dolphin can consume between 8 and 15 kg of fish per day, there is a potential for significant contamination to occur through trophic transfer.

Given its semi-enclosed basin, high anthropogenic river inputs, and densely populated coasts, the Black Sea is heavily impacted by pollution and litter accumulation [54,61,62,63,64]. The high amount of microplastics in all Black Sea compartments exposes the organisms that live there to plastic pollution. The high plastic contamination in the Black Sea could be highlighted also by the high amount of microplastics found in this study. However, it should be mentioned that the relatively short duration (1 year) of this study, along with the limited number of stranded and by-caught cetaceans, has led to the analysis of a small sample size. Due to these constraints, further studies are required to provide a more comprehensive overview of microplastic pollution in Black Sea cetaceans. Nevertheless, the findings will constitute a significant foundational framework and comparative reference for future investigations in this underexplored domain within the Black Sea region.

Based on these first results, we may state that the monitoring of microplastics in the GIT of Black Sea cetaceans, under the MSFD, could provide valuable insights into this threat. 

As top predators, the level of microplastic contamination in the GIT of cetaceans provides valuable insights into adjacent trophic levels. Long-term monitoring can bring crucial information for an inaccessible and understudied area (the water-sediment interface), considering that certain cetacean species primarily consume benthic organisms [34]. Furthermore, employing a methodology in line with the MSFD, as utilized in this study, can offer important data for the implementation of this European policy. 

Certainly, this approach faces limitations, primarily associated with the collection and analysis of a sufficiently large number of samples to establish thresholds and ultimately assess the ecological status of the marine environment according to D10-marine litter criteria. Another crucial factor that could pose challenges is secondary contamination, particularly when handling large samples such as an entire gastro-intestinal tract (GIT) [28]. In our study, efforts were made to eliminate all possible contamination sources; however, in cases where removal was not feasible, such as the white nylon serrated band used for GIT sealing, white safety gloves, and a green hose utilised during GIT washing, their respective colours were noted. This approach allowed us to assess their potential impact on the final results. Subsequent analysis revealed the presence in the samples of only one white particle and six green particles. Green particles were not found in procedural blanks and controls. Even after removing primary contamination sources, procedural blanks and controls remained essential to control secondary contamination during the study. Procedural blanks were taken before washing the GIT, and control samples were maintained throughout the activity. All particles found (*n* = 18) in procedural blanks and controls were removed from the study’s database in a 1:1 ratio to uphold data integrity [30].

Nevertheless, with the effective management of research efforts and strengthened collaboration with the fishing sector and with competent authorities, these limitations could be minimised. 

## 5. Conclusions

Cetaceans, positioned at the top of the marine food chain, play a pivotal role in reflecting the nowadays issue of plastic pollution in the Black Sea. Microplastics and mesoplastics were ingested by all analysed individuals in this study. Broadly, our observations on plastic items’ colour and form align with the outcomes of prior research. In all samples, microplastics dominated numerically, being much higher than reported in all relevant worldwide studies. This first report of the highest incidence of ingested microplastics in cetaceans could be the consequence of variations in the sample processing methodologies and, more particularly, the level of microplastics in the Black Sea waters, which are considered to be the most plastic-polluted within Europe [65]. Further efforts are required to collect additional data and to harmonize and implement a standardized protocol for the processing of cetacean GIT samples at the regional or even European level.

## Figures and Tables

**Table 1:** General information on investigated individuals.

Animal ID	Species	Coordinates	Found	Estimated Age	Sex	DCC	Organ
PCETSTR040423#1	*T. t. ponticus*	44.0390 28.6515	Stranded	Adult	Female	2	Stomach Intestine
PCETSTR180423#2	*T. t. ponticus*	44.4323 28.6455	Stranded	Adult	Male	3	Stomach Intestine
PCETGN140223#1	*T. t. ponticus*	44.4153 29.4459	By-caught	Adult	Male	2	Stomach Intestine
PCETGN090423#2	*P. p. relicta*	44.3527 29.1578	By-caught	Adult	Male	2	Stomach Intestine

## Data Availability

The detailed report and the data will be available on the ACCOBAMS website.
